# A promoter region of the midkine gene that is frequently expressed in human hepatocellular carcinoma can activate a suicide gene as effectively as the *α*-fetoprotein promoter

**DOI:** 10.1038/sj.bjc.6601246

**Published:** 2003-09-09

**Authors:** M Tomizawa, L Yu, A Wada, T Tamaoki, K Kadomatsu, T Muramatsu, S Matsubara, K Watanabe, M Ebara, H Saisho, S Sakiyama, M Tagawa

**Affiliations:** 1 Division of Pathology, Chiba Cancer Center, 666-2, Nitona, Chuo-ku, Chiba 260-8717, Japan; 2 Department of Medicine and Oncology, Graduate School of Medicine, Chiba University, 1-8-1 Inohana, Chuo-ku, Chiba 260-8670, Japan; 3 Department of Respirology, Graduate School of Medicine, Chiba University, 1-8-1 Inohana, Chuo-ku, Chiba 260-8670, Japan; 4 Department of Medical Biochemistry, University of Calgary, 3330 Hospital Drive NW, Calgary, Alberta, Canada T2N4 N1; 5 Department of Biochemistry, Nagoya University, School of Medicine, 65 Tsurumai-cho, Showa-ku, Nagoya 466-8550, Japan; 6 Department of Biochemistry, Faculty of Medicine, Kagoshima University, 8-35-1 Sakuragaoka, Kagoshima 890-8520, Japan; 7 Division of Gastroenterological Surgery, Chiba Cancer Center, 666-2, Nitona, Chuo-ku, Chiba 260-8717, Japan

**Keywords:** midkine, promoter, hepatocellular carcinoma, *α*-fetoprotein, HSV-TK

## Abstract

We examined the expression of the *midkine* (*MK*) and *α-fetoprotein* (*AFP*) genes in 15 paired human specimens obtained from hepatocellular carcinoma (HCC) and the corresponding noncancerous regions of the same patients. A total of 14 HCC but none of the noncancerous specimens were positive for the *MK* mRNA. In contrast, three HCC specimens and one corresponding noncancerous sample out of the three AFP-positive HCC cases expressed the *AFP* gene. A 2.3-kb genomic fragment in the regulatory region of the *MK* gene could activate a fused reporter gene in both AFP-producing and -nonproducing HCC lines, and the MK fragment-mediated transcriptional activity was comparable to the AFP enhancer-linked AFP promoter in AFP-producing cell lines. The AFP-producing but not AFP-nonproducing HCC cell lines that were transfected with the MK promoter-linked *herpes simplex virus-thymidine kinase* (*HSV-TK*) gene became susceptible to a prodrug ganciclovir to a similar degree of the HCC transfected with the enhancer-linked AFP promoter-fused *HSV-TK* gene. These data suggest that the MK promoter can activate a therapeutic gene preferentially in HCC and is as useful as the AFP promoter in clinical settings.

Preferential expression of a cytotoxic gene in tumours is a therapeutic approach to cancer treatment. Regulatory sequences of the gene that is expressed primarily in tumours can activate a fused exogenous gene in the tumours and the use of such promoter regions has been investigated for its clinical feasibility (Miller and Whelan, 1997). Regulated expression of a suicide gene with the promoters can primarily destroy tumour cells and leave the surrounding tissues undamaged. The *α*-fetoprotein promoter (AFP) is a representative one to activate an exogenous gene specifically in hepatocellular carcinoma (HCC) (Tamaoki, 2000) and has been applied to targeted gene therapy for HCC (Kanai *et al*, 1996; Mawatari *et al*, 1998; Hallenbeck *et al*, 1999). Precise analysis of the regulatory region of the *AFP* gene demonstrated that enhancer and silencer elements in the region played a crucial role in the transcription (Nakabayashi *et al*, 1991) and the silencer-deleted, enhancer-linked AFP promoter showed noticeable therapeutic effects compared with the unmodified AFP promoter (Mawatari *et al*, 1998).

Midkine (MK) is a heparin-binding growth factor (Kadomatsu *et al*, 1988) with a number of functions: forced expression of MK induced transformation (Kadomatsu *et al*, 1997) and elevated angiogenesis (Choudhuri *et al*, 1997). Expression of the *MK* gene in human adult tissues is extremely low and restricted; however, the expression is upregulated in a number of human tumours particularly in gastrointestinal tumours including HCC (Aridome *et al*, 1995; Koide *et al*, 1999). The 5′ upstream region of the *MK* gene was demonstrated to activate a suicide gene in MK-positive human tumours (Adachi *et al*, 2000; Miyauchi *et al*, 2001; Wesseling *et al*, 2001). In this study, we compared the usefulness of the AFP and the MK promoters for HCC treatment by examining the frequency of *MK* and *AFP* expression in human HCC specimens and the transcriptional activity of the promoters in HCC cell lines.

## MATERIALS AND METHODS

### Tissue samples and cells

Paired cancerous and noncancerous specimens obtained from the same HCC patients were provided by the Chiba Cancer Center Tissue Bank. The clinical data regarding respective patients were provided by the Bank based on the ethical guideline on human genome study and genetic analysis (issued by the Japanese government in 2001). Histological types of HCC were determined according to the classification of the Liver Cancer Study Group of Japan. Human HCC cells (HuH-7, PLC/PRF/5, HLE and HLF cells), human breast cancer MCF-7 cells and human pancreatic cancer AsPC-1 cells were cultured in Dulbecco's modified Eagle's or RPMI1640 medium supplemented with 10% fetal calf serum.

### Northern blot analysis

Total RNA (10 *μ*g) was isolated with Trizol (Life Technologies, Rockville, MD, USA), subjected to electrophoresis in a denaturing formaldehyde–agarose gel and transferred to a nylon filter. MK (Kadomatsu *et al*, 1988) and AFP cDNA (Morinaga *et al*, 1983) were labelled with [^32^P]dCTP and used as probes. The hybridisation was performed in a solution of 50% formamide/5 × SSC/50 mM NaH_2_PO_4_/2 × Denhardt's solution/0.1% SDS/0.1 mg ml^−1^ salmon sperm DNA at 42°C for 12 h. The filter was washed with a solution of 0.2 × SSPE/0.1% SDS several times at 50°C. The same filter was rehybridised with a human 18S ribosome cDNA as a control.

### Dual luciferase assay

The 2335-bp genomic DNA fragment of the *MK* gene (−2285/+50, +1 corresponds to the transcription start site) (Uehara *et al*, 1992) was cloned into the pGL2-basic vector (Promega, Madison, WI, USA) that contained the *firefly luciferase* gene without any promoter sequences (MK2.3-luc). MK2.3kb-luc DNA was digested with *Xho*I/*Eco*47III to produce the 609-bp fragment-linked *firefly luciferase* genes (MK0.6-luc). The AFP promoter region (AFP0.2, 212-bp *Sac*I/*Hind*III fragment) and the enhancer (934-bp *Swa*I/*Eco*RI fragment)-linked AFP promoter region (AFPEn0.2) were prepared from pAF5.1-CAT (Nakabayashi *et al*, 1991), and they were subcloned into pGL2-basic vector (AFP0.2-luc, AFPEn0.2-luc). The transcriptional activity was measured with the dual luciferase reporter assay system (Promega). Plasmid DNA containing respective genomic fragments, pGL2-control vector (Promega) harbouring the SV40 promoter-linked *firefly luciferase* gene (SV40-luc) or pGL2-basic, together with a control vector, the *renilla luciferase* gene fused with the HSV-TK promoter (pRL-TK, Promega) at a molar ratio of 10 : 1, was transfected into target cells with a lipofectin reagent (Life Technologies, Gaithersburg, MD, USA). After 2 days, cells were lysed and the luciferase activities were measured according to the manufacturer's protocol. The relative firefly luciferase activity of each cell lysate was calculated based on the amount of luminescence produced by renilla luciferase and was expressed as a percentage of the SV40 promoter-mediated activity. All of the values were expressed as a mean of four independent experiments. The statistical analysis was performed by one-way analysis of variance (ANOVA).

### *In vitro* sensitivity to ganciclovir

The 2.3 kb. MK or the enhancer-linked AFP promoter region was ligated into pcDNA3 vector (Invitrogen, San Diego, CA, USA) from which the cytomegalovirus promoter was removed. The *herpes simplex virus-thymidine kinase (HSV-TK)* gene was then cloned into the downstream of the promoters (MK2.3-TK, AFPEn0.2-TK). Cells were transfected with MK2.3-TK, AFPEn0.2-TK or pcDNA3 vector DNA and G418 (Life Technologies)-resistant cells were selected. The pooled cells were seeded in 96-well plates at a density of 5 × 10^3^ cells well^−1^ and were cultured with various concentrations of ganciclovir (GCV). After 5 days, viable cells in each well were measured with a cell-counting kit (Wako, Osaka, Japan). The amount of formazan produced in each well was determined from the absorbance at 450 nm.

## RESULTS

### Expression of the *MK* and *AFP* genes in HCC

We examined the frequency of expressed *MK* and *AFP* genes in paired HCC and noncancerous specimens of the same patients by Northern blot analysis. In all, 14 out of 15 HCC specimens but none of the corresponding noncancerous samples was positive for *MK* mRNA expression. Three HCC specimens expressed the *AFP* gene and one of the *AFP*-positive samples was also *AFP*-positive in the corresponding noncancerous region (Table 1

**Table 1:** Expression of the *MK* and *AFP* genes in human HCC specimens

					**Pathology**		**Expression^a^**		
					**HCC**	**Noncancerous**	**MK**	**AFP**	**Serum AFP^b^**
**No**	**Age**	**Gender**	**HBs antigen**	**HCV antibody**			**(HCC/Noncancerous)**		**(ng ml^−1^)**
1	23	M	+	−	mode, trab/pseud	CH	+/−	+/+	21
2	48	M	+	−	mode, sci	CH	+/−	+/−	10 892
3	52	M	+	−	mode, trab	CH	+/−	−/−	70
4	57	M	−	+	mode, trab	CH	+/−	−/−	23
5	61	M	−	+	well, trab	CH	+/−	−/−	15
6	66	M	−	+	poor, com	CH	+/−	−/−	5
7	68	M	−	+	mode, trab	CH	+/−	−/−	3
8	69	M	+	−	mode, trab	CH	+/−	−/−	26
9	73	M	+	−	mode, trab	CH	+/−	−/−	124
10	49	F	+	−	mode, trab	CH	+/−	−/−	3
11	52	F	NA	NA	poor, com	CH	−/−	−/−	41
12	58	F	+	−	mode, trab	CH	+/−	+/−	1434
13	59	F	−	+	well, trab	CH	+/−	−/−	572
14	74	F	−	+	mode, trab	LC	+/−	−/−	2999
15	55	M	−	+	well, trab	LC	+/−	−/−	14

Abbreviations: M=male, F=female, NA=not available, well=well differentiated, mode=moderately differentiated, poor=poorly differentiated, trab=trabecular type, pseu=pseudoglandular type, com=compact type, sci=scirrhous type, CH=chronic hepatitis, LC=liver cirrhosis.

a Expression of the *MK* and *AFP* genes in HCC and noncancerous region of the same patients was examined with Northern blot analysis and was shown as positive (+) or negative (−).

b Serum AFP before surgical operation.

, Figure 1

**Figure 1:**
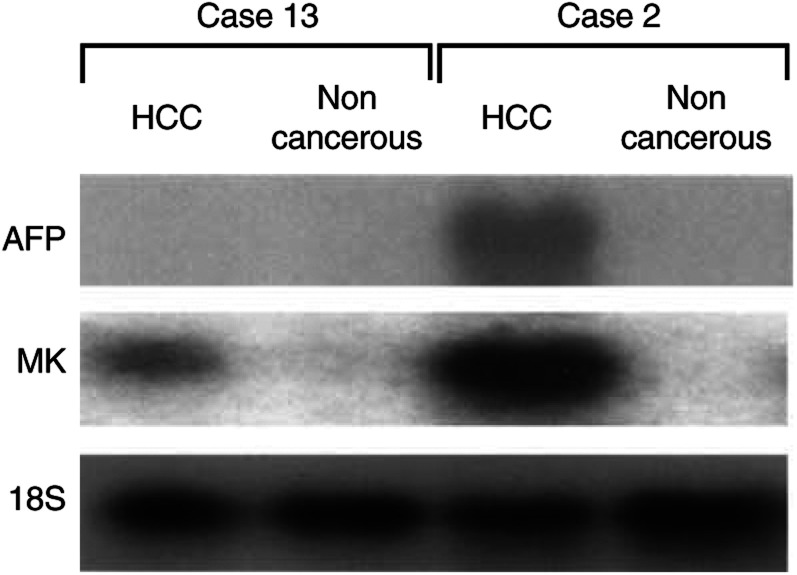
Expression of the *MK* and *AFP* genes in human surgical specimens was examined with Northern blot analysis. Representative samples were shown and the expression of *18S ribosomal RNA* gene was used as controls.

). Frequency and tumour specificity of the expression in human HCC was thus greater for the *MK* compared with the *AFP* gene. Serum AFP values of the patients were not correlated with the results of *AFP* mRNA expression.

### Transcriptional activity of MK and AFP promoter

We investigated the transcriptional activity of MK and AFP promoters with the luciferase reporter assay. Our previous studies showed that a 2.3- or a 0.6-kb genomic fragment of the *MK* gene contained *cis*-acting elements, which could activate an exogenous gene preferentially in tumours (Miyauchi *et al*, 2001; Yoshida *et al*, 2002). Linkage of 0.2-kb AFP promoter and 0.9-kb AFP distal enhancer gave the maximal transcription of a fused reporter gene (Nakabayashi *et al*, 1991). We thereby examined the transcriptional activity of four kinds of reporter constructs (MK2.3-luc, MK0.6-luc, AFP0.2-luc and AFPEn0.2-luc) in AFP-producing (HuH-7 and PLC/PRF/5) and -nonproducing (HLE and HLF) HCC cell lines (Niwa *et al*, 1996; Ishikawa *et al*, 1999), and in non-HCC cell lines (MCF-7 and AsPC-1), which were positive for MK expression (Miyauchi *et al*, 2001).

**Figure 2:**
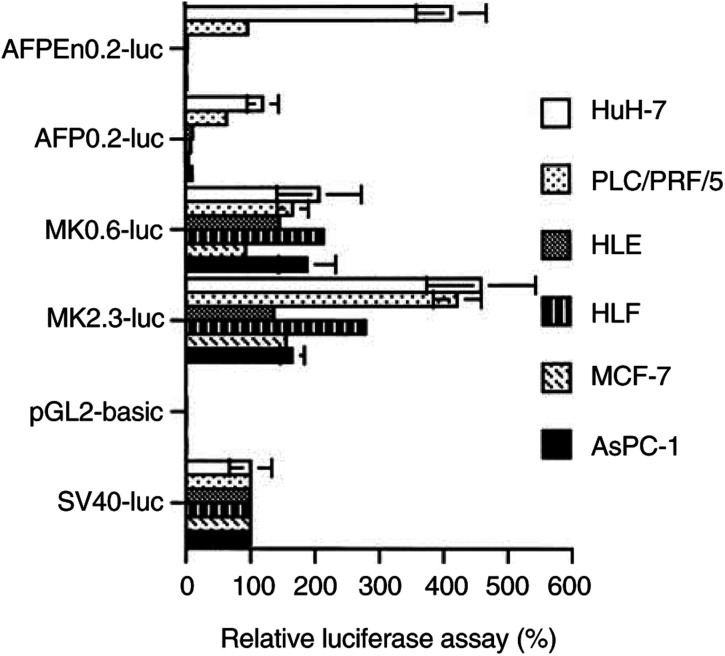
Transcriptional activity of the MK and AFP genomic fragments tested in AFP-producing HCC (HuH-7 and PLC/PRF/5), AFP-nonproducing HCC (HLE and HLF) and non-HCC (MCF-7 and AsPC-1) cells. The relative firefly luciferase activity was expressed as a percentage of the SV40 promoter-mediated activity. Standard error bars are also shown.

Introduction of the respective reporter genes into HCC cell lines revealed that transcriptional activity of the 2.3- or 0.6-kb MK fragment was greater than that of the SV40 or the AFP promoter (*P*<0.001, Figure 2

). Since HuH-7 and PLC/PRF/5 cells are AFP-high and AFP-intermediate producers, respectively (Niwa *et al*, 1996; Ishikawa *et al*, 1999), the transcriptional activity of the AFP promoter was greater in HuH-7 than in PLC/PRF/5 cells. Addition of AFP enhancer augmented the AFP promoter-mediated transcriptional activation particularly in HuH-7 cells (*P*<0.001). The luciferase activity of AFPEn0.2-luc DNA was then comparable to that of the MK2.3-luc DNA in HuH-7 cells. In AFP-nonproducers, however, the transcriptional activity of the AFP promoter or the enhancer-linked AFP promoter was minimal.

The transcriptional activity of the 2.3- or 0.6-kb MK fragment was comparable to that of the SV40 promoter in non-HCC cells as previously reported (Miyauchi *et al*, 2001) and the AFP promoter did not activate the fused luciferase gene in these cells.

### *In vitro* sensitivity to GCV

**Figure 3:**
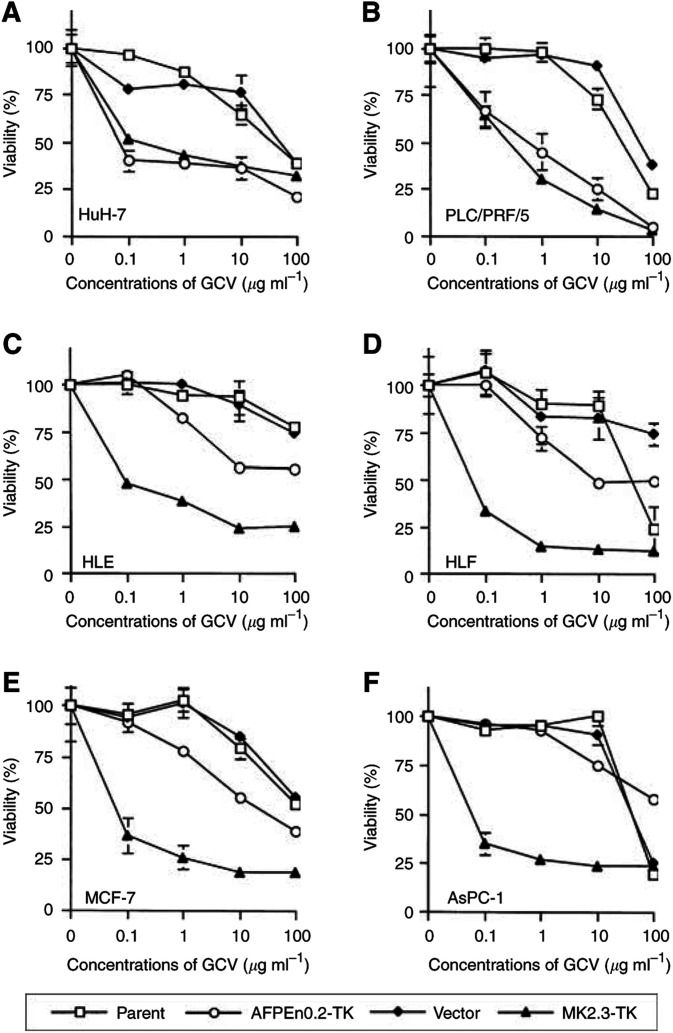
Susceptibility of HuH-7 (**A**), PLC/PRF/5 (**B**), HLE (**C**), HLF (**D**), MCF-7 (**E**) and AsPC-1 (**F**) cells to various concentrations of GCV. Parent cells and G418-resistant cells that were transfected with vector, MK2.3-TK or AFPEn0.2-TK DNA were used. Standard error bars are also shown.

We compared increased cytotoxic activity caused by the HSV-TK/GCV system using the MK and the AFP promoters. All the cells were transfected with MK2.3-TK, AFPEn0.2-TK or vector DNA and then respective G418-resistant cells were exposed to various concentrations of GCV (Figure 3

). The sensitivity of vector DNA-transfected cells was not different from that of respective parent cells. However, AFP-producing HuH-7 and PLC/PRF/5 cells increased the susceptibility to GCV, when they were transfected with MK2.3- or AFPEn0.2-TK DNA (Figure 3A and B). PLC/PRF/5 cells were more susceptible to GCV than HuH-7 cells, although the transcriptional activity of both MK and AFP promoters was stronger in HuH-7 than in PLC/PRF/5 cells. AFP-nonproducing HCC and non-HCC cells that were transfected with MK2.3-TK DNA significantly increased their susceptibility to GCV and those transfected with AFPEn0.2-TK DNA also slightly increased the susceptibility (Figure 3C–F). The increased sensitivity of the AFPEn0.2-TK transfectants, despite minimal transcriptional activity of the AFPEn0.2 DNA in these cells, was probably due to multiple integration of transfected DNA, in which condition cellular and/or plasmid-derived promoters could activate the *TK* gene.

## DISCUSSION

In this study, we showed that human HCC specimens expressed the *MK* gene more frequently than the *AFP* gene and that the transcriptional activity of the MK promoter was as strong as that of the enhancer-linked AFP promoter. The present data also indicate that the MK promoter could activate the *HSV-TK* gene in HCC cells to a similar extent as the AFP promoter. The AFP promoter has been used to activate a suicide gene in HCC cell lines (Kanai *et al*, 1996; Mawatari *et al*, 1998), to produce a hybrid promoter in combination with other regulatory regions (Ido *et al*, 2001) and to apply to oncolytic adenovirus (Hallenbeck *et al*, 1999). These previous studies suggested the feasibility of the AFP promoter-mediated gene therapy for HCC. However, relatively low frequency of *AFP* expression in human HCC may hamper the clinical application. Although serum AFP was elevated in 70% of HCC patients (Nakabayashi *et al*, 1991), AFP transcripts were not frequently detected with Northern blot analysis as shown in the present study. Di Biscegile *et al*. (1986) reported that nine out of 10 HCC patients showed elevated serum AFP values, but only two out of the 10 HCC specimens were positive for the mRNA with Northern blot analysis. Similar cases, high serum AFP values with undetectable AFP transcript with Northern blot analysis, were also reported (Varagona *et al*, 1992; Niwa *et al*, 1996), although the level of *AFP* mRNA expression in general correlated with serum AFP levels (Niwa *et al*, 1996). With more sensitive methods, noncancerous samples as well as HCC specimens turned out to be positive for *AFP* mRNA expression, since preneoplastic cells in liver cirrhosis could produce AFP (Otsuru *et al*, 1988). These data collectively raise the possibility that the AFP promoter may not activate fused exogenous genes in some of serum AFP-positive HCC cases. On the contrary, frequent expression of the *MK* gene implies a possible strategy of MK promoter-based therapy for HCC patients, although the expression is not specific to HCC.

The specificity of transcriptional activity by the MK and the AFP promoter has been already demonstrated; both promoters could drive the fused gene in respective MK- or AFP-positive cells but not in MK- or AFP-negative cells (Kanai *et al*, 1996; Miyauchi *et al*, 2001). The mechanism of preferential expression of the *MK* gene in tumours was not fully understood. It could be related with cell growth: forced expression of MK in fibroblasts induced cell transformation (Kadomatsu *et al*, 1997) and suppressed expression of MK inhibited the tumour growth (Takei *et al*, 2001).

Recent studies demonstrated feasible application of tumour-specific promoters to oncolytic adenovirus, which could replicate within tumours but not in normal tissues (Heise and Kirn, 2000). The MK promoter-mediated oncolytic adenovirus was not harmful to liver but killed MK-positive tumours (Adachi *et al*, 2000). These data suggested that MK promoter-based gene therapy could facilitate selective killing of HCC and keep surrounding noncancerous tissues undamaged. The present Northern blot analysis, in fact, implied that MK promoter-mediated gene therapy for HCC would not injure surrounding cirrhosis tissues. Extracorporal intratumoural injection technique could also broaden the application of MK promoter-based therapy for HCC patients. Since maintenance of residual liver functions is crucial for HCC treatment, the MK promoter could provide an alternative therapeutic approach besides AFP promoter-based therapy. The present study suggested that the AFP promoter and the MK promoter are applicable to AFP-high- and AFP-low/nonproducing HCC, respectively, and the feasibility of both kinds of the promoter-based therapy will be investigated in clinical settings.
